# Oocyte aging-induced *Neuronatin* (*NNAT*) hypermethylation affects oocyte quality by impairing glucose transport in porcine

**DOI:** 10.1038/srep36008

**Published:** 2016-10-26

**Authors:** Ying-Ying Gao, Li Chen, Tao Wang, Zheng-Wen Nie, Xia Zhang, Yi-Liang Miao

**Affiliations:** 1 Key Lab of Agricultural Animal Genetics, Breeding, and Reproduction of Ministry of Education, College of Animal Science and Technology, Huazhong Agricultural University, Wuhan, 430070, China; 2 College of Veterinary Medicine, Huazhong Agricultural University, Wuhan 430070, China; 3 The Cooperative Innovation Center for Sustainable Pig Production, Wuhan 430070, China

## Abstract

DNA methylation plays important roles in regulating many physiological behaviors; however, few studies were focused on the changes of DNA methylation during oocyte aging. Early studies showed that some imprinted genes’ DNA methylation had been changed in aged mouse oocytes. In this study, we used porcine oocytes to test the hypothesis that oocyte aging would alter DNA methylation pattern of genes and disturb their expression in age oocytes, which affected the developmental potential of oocytes. We compared several different types of genes and found that the expression and DNA methylation of *Neuronatin* (*NNAT*) were disturbed in aged oocytes significantly. Additional experiments demonstrated that glucose transport was impaired in aged oocytes and injection of NNAT antibody into fresh oocytes led to the same effects on glucose transport. These results suggest that the expression of *NNAT* was declined by elevating DNA methylation, which affected oocyte quality by decreasing the ability of glucose transport in aged oocytes.

The quality of oocyte is critical for the development of embryos after fertilization. However, the quality of oocytes would be decreased during oocyte aging, either *in vivo* aging or *in vitro* aging^1^. In mammals, oocyte aging has been found to lead to parthenogenesis^2^, increased susceptibility to activating stimuli^3^ and abnormal and/or retarded development of embryos/fetuses^4^. Delaying oocyte manipulation is common in many researches, animal reproductive technologies and clinic assisted reproduction technologies (ART). In mammals, it is very important and necessary to study mechanisms underlying oocyte aging, which will have advantages to control oocyte aging and provide more time to manipulate oocyte.

DNA methylation plays important roles in regulating many physiological behaviors. Establishment and maintenance of DNA methylation of specific genes in oocytes are part of the maturation process of oocytes and essential for normal development after fertilization. Imamura *et al*.^5^ reported *Peg1/Mest* became hypermethylated after oocytes were cultured *in vitro* for short time, whereas prolonged *in vitro* culture resulted in demethylation in a fraction of mouse oocytes^5^. Our previous data showed that *Snrpn* was fully methylated in fresh oocytes and the methylation would be lost at 29 h post-hCG both in *in vivo* aged oocytes and *in vitro* aged oocytes without cumulus cells in mouse^6^. However, oocyte aging caused a decline in reproductive outcomes but did not evidently lead to defects in DNA methylation imprinting acquisition in the oocytes from viable offspring^7^.

Glucose metabolism affected both oocyte maturation and following development of oocytes after fertilization and oocyte aging^8^,^9^. Glucose metabolism in cumulus cells prevented oocyte aging by producing pyruvate and NADPH through glycolysis and pentose phosphate pathway (PPP). Lactate prevented oocyte aging mainly by producing NADH (through its lactate dehydrogenase-catalyzed oxidation to pyruvate), which would then be converted into ATP through mitochondrial electron transport. However, pyruvate did not rely solely on electron transport for its inhibition of oocyte aging. Both pyruvate and lactate involved mitochondrial electron transport and monocarboxylate transporters (MCTs) were active on the plasma membrane and/or mitochondria of the aging oocyte. Pyruvate regulated both the intracellular redox status and energy supply at a higher concentration but regulated only energy supply at a lower concentration to inhibit oocyte aging^9^. Well-balanced and timed glucose metabolism need enough and timely glucose transport in oocytes^10^. It was found that NEURONATIN (NNAT) was an important protein to regulate glucose transport 1(GLUT-1) by activating PI3K-Akt2 signaling pathway^11^. *NNAT* was a maternal imprinted gene and changes in DNA methylation caused the maternal allele to lose imprinting and trigger cell proliferation and metastasis^12^. NNAT took roles in neuronal differentiation in the brain^13^ and increased insulin secretion by regulating intracellular calcium levels and hyperglycemia-induced apoptosis in pancreatic β-cells^14^. In porcine placenta, *NNAT* was monoallelically expressed and regulated glucose transporter genes^15^.

It is necessary to understand the mechanisms of oocyte aging, which will be beneficial to find methods to avoid oocyte aging. However, few studies were focused on the dynamics of DNA methylation during oocyte aging. We therefore proposed a hypothesis that oocyte aging would alter DNA methylation pattern of some important genes and disturb their expression, which would change some related signaling pathways and affect the development of embryos after fertilization. Besides imprinted genes, maternal genes and pluripotent genes are important for oocyte-to-embryo transition (OET) and following development after fertilization in oocytes. We also selected several important maternal genes and pluripotent genes for detection. To test this hypothesis, we used porcine oocytes aging *in vitro* as model and selected several important imprinted genes, maternal genes and pluripotent genes and compared their expression in fresh and aged porcine oocytes. Then we tried to analyze their DNA methylation pattern of genes with abnormal expression and physiologic effects in aged oocytes.

## Materials and Methods

Chemicals and reagents used in the present study were purchased from Sigma Chemical Co. unless otherwise specified.

### Preparation of porcine oocytes

Porcine ovaries were obtained from a slaughterhouse and transported to the laboratory while maintained at <34 °C. Follicular fluid from 3–6 mm antral follicles was aspirated with an 18-gauge syringe. Cumulus oocyte complexes (COCs) with uniform cytoplasm and several layers of cumulus cells were selected and rinsed three times in washing medium (TCM-199 medium supplemented with 10% porcine follicular fluid (pFF), 5 μg/mL insulin, 10 ng/mL EGF, 0.6 mM cysteine, 0.2 mM pyruvate, 25 μg/mL kanamycin). Approximately 30 COCs per well were cultured in 96 well plates containing TCM-199 medium supplemented with 10% porcine follicular fluid (pFF), 5 μg/mL insulin, 10 ng/mL EGF, 0.6 mM cysteine, 0.2 mM pyruvate, 25 μg/mL kanamycin and 5 IU/mL of each eCG, and hCG, covered with mineral oil. The oocytes were matured for 44 h at 38.5 °C, 5% CO_2_ in humidified air.

### *In vitro* aging of porcine oocytes

For denuded oocytes (DO) aging *in vitro*, cumulus cells were removed by vortexing for 4 min in 0.1% hyaluronidase (in TLH-PVA^16^, TL-Hepes medium (TLH, 114.0 mM NaCl, 3.1 mM KCl, 2.0 mM CaCl_2_.2H_2_O, 0.5 mM MgCl_2_.6H_2_O, 0.3 mM NaH_2_PO_4_, 10 mM Na-Lactate, 0.25 mM Na-pyruvate, 2.0 mM NaHCO_3_, 10.0 mM HEPES, 0.075 mg/mL Kanamycin, 0.015 mg/mL Phenol) supplemented with 0.1% PVA) after porcine COCs maturated for 44 h. Only oocytes with first polar bodies were used for the experiments. For COC aging *in vitro*, cumulus cells would not be removed. The treated oocytes were then cultured in wells of a 96-well culture plate containing 150 μl of NCSU23 medium (108.7 mM NaCl, 4.8 mM KCl, 1.7 mM CaCl_2_.2H_2_O, 1.2 mM KH_2_PO_4_, 1.2 mM MgSO_4_.7H_2_O, 25.1 mM NaHCO_3_, 5.5 mM Glucose, 1.0 mM L-Glutamine, 7.0 mM Taurine, 5.0 mM Hypotaurine, 0.05 mg/mL Gentamicin, 4.0 mg/mL Fatty acid-free BSA)^17^ and covered with mineral oil at 38.5 °C under 5% CO_2_ in humidified air for 24 or 48 h.

### RNA isolation and real-time RT-PCR

Total RNA was isolated from 50 porcine oocytes or blastocyts using an Arcturus Pico Pure kit (Life Technologies, Grand Island, NY). Enhanced GFP (eGFP) cRNA was transcribed *in vitro* from *pIVT-eGFP*^18^ and 1 ng was added to each sample prior to RNA isolation as an internal control. Real-time RT-PCR was performed as previously described^19^, using cDNA from two oocytes or embryos per reaction. Relative gene expression was calculated using the ΔCt method^20^ with *eGFP* expression for normalization. Primers were listed in Table S1.

### Parthenogenetic activation and *in vitro* culture of embryos

Fresh or aged oocytes were placed between 0.2-mm-diameter platinum electrodes 1 mm apart in activation medium. Activation was induced with two direct-current (DC) pulses of 1.2 kV/cm for 40 μs on a BTX Elector-Cell Manipulator 200 (BTX, San Diego, CA) according to the experimental design. The medium used for activation was 0.3 M mannitol, supplemented with 1.0 mM CaCl2, 0.1 mM MgCl2, and 0.5 mM Hepes. The orientation of oocytes and polar bodies was not vertical to platinum wire electrodes during electrical activation. After activation treatment, embryos were washed and transferred into NCSU medium with 5 μg/ml cytochalasin B for 4 h to inhibit second polar body extrusion, then cultured in 150 μl NCSU medium covered with mineral oil in a 96-well culture plate. The culture environment was 5% CO_2_ in air at 38.5 °C. Parthenogenetically activated oocytes were evaluated for the blastocyst percentage on Day 6.

### PCR amplification, cloning and sequencing

Oocyte or blastocyst genomic DNA was modified using the EZ DNA Methylation-Direct Kit (Zymo Research) according to the manufacturer’s instructions. The following steps were conducted according to our previous report with minor modifications^21^. To obtain PCR products, two individual nested PCRs were carried out using 2 μl bisulfite-treated DNA in the first round PCR of 25 μl reaction system and 2 μl of the first round PCR products as templates in the second round PCR of 50 μl reaction system. All reactions contained 0.4 mM primers, 0.2 mM dNTP, 50 mM KCl, 10 mM Tris–HCl, 1.5 mM MgCl2, and 1.25 U of rTaq Hotstart polymerase (TaKaRa, Japan). The PCRs were performed with a Bio-Rad T100 (USA) using the following programs. The program for the first round was 1 cycle at 94 °C for 6 min; 35 cycles of 94 °C for 1 min, 50 °C for 1 min, 72 °C for 3 min; and 1 cycle of 72 °C for 10 min. For the second round PCR, the program was 1 cycle at 94 °C for 4 min; 30 cycles at 94 °C for 1 min, 56 (*NNAT*) or 55 °C (*H19*) for 1 min, and 72 °C for 1 min, and 1 cycle at 72 °C for 10 min. Products of the second round PCR were then recovered and gel-purified using the Universal DNA purification Kit (Tiangen, China). Purified fragments were subcloned into T-vectors (TaKaRa, Japan). The clones confirmed by PCR were selected for DNA sequencing using an automatic sequencer (ABI Prism-77). Three independent amplification experiments were carried out for each treatment. We sequenced around ten clones from each independent set of amplification and cloning. Primers were listed in Table S2.

### Analysis by COBRA

Half of all purified PCR products used for cloning and sequencing from the three repeats in each treatment were pooled together and some of them were digested with restriction enzyme BstU I (NEB, USA). The digested fragments were electrophoresed on 2.5% agarose gels.

### Evaluation of glucose transport in oocytes

2-NBDG (Sigma, Cat# 72987), a fluorescent glucose analog, was used to track glucose transport. In brief, oocytes were incubated in TLH-PVA with 200 μM 2-NBDG for 40 min. Following three rapid washes, live cells were immediately imaged at 488 nm by fluorescence microscope (Olympus, BX53, Japan). Fluorescence signal was quantified using NIH Image J software and then was calculated as the average intensity after background subtraction.

### Fluorescence and Immunofluorescence (IF) Microscopy

Zona pellucida of oocytes was removed with 0.25% pronase, washed in PBS, and then fixed in 4% (W/V) paraformaldehyde in PBS for 1 hr at room temperature (RT). Oocytes were washed three times in PBS and then placed into 50% methanol for 5 min, 100% methanol for 5 min, 100% acetone for 5 min to extract lipid droplets that are abundant in porcine oocytes. Lipid-extracted oocytes were rehydrated and permeablized in 1% Triton X-100 (V/V) permeabilization solution (1% Triton X-100, 20 mM Hepes, pH 7.4, 3 mM MgCl2, 50 mM NaCl, 300 mM sucrose, 0.02% NaN3 in PBS) for 1 hr at RT. After oocytes had been blocked with 3% BSA for 1 hr at RT, they were stained with NNAT antibody (Bioss, Cat# bs-11519R, Beijing), washed three times, stained with anti-rabbit IgG second antibodies at a dilution of 1:1000 for 1 hr at RT. The oocytes were counterstained with 1 mg/ml DAPI in Vectashield mounting medium (Vector Laboratories, Burlingame, CA) to stain DNA. Finally, oocytes were mounted on glass slides. Slides were scanned by using a Zeiss confocal microscope (Zeiss LSM 510 UV). At least 30 samples in each group were analyzed in three repeated experiments.

### Antibody injection

About 5–10 pl NNAT antibody was microinjected into the cytoplasm of a fresh MII oocyte using a Nikon Ti-S inverted microscope (Nikon, Japan) equipped with FemtoJet^®^ 4i (Eppendorf, Germany). After microinjection, the oocytes were cultured for 5 h in NCSU medium under paraffin oil at 38.5 °C, in an atmosphere of 5% CO_2_ in air. Oocytes in control group were microinjected with 5–10 pl rabbit immunoglobulin G (IgG) of the same concentration.

### Data Analysis

For each treatment, three replicates were run. Statistical analyses were carried out by analysis of variance. Differences between control and treated aged groups were evaluated with the Duncan multiple comparison test. Data are expressed as mean ± SEM and P < 0.05 is considered significant.

## Results

### *NNAT* was downregulated in aged oocytes and blastocysts by parthenogenetic activation

**Figure 1:**
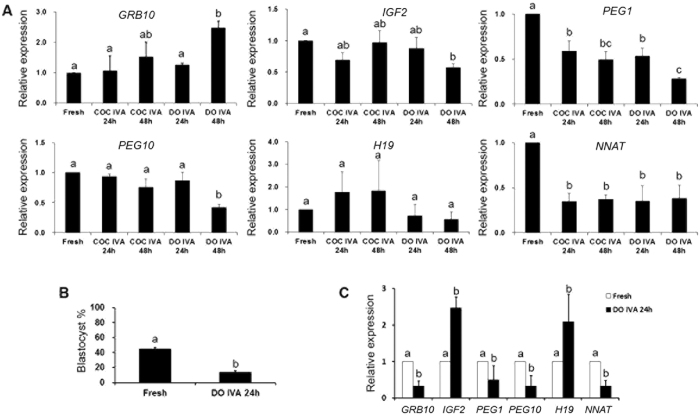
The expression of related imprinted genes in fresh and aged oocytes and blastocysts by parthenogenetic activation. (**A**) Relative expression of related imprinted genes (*GRB10, IGF2, PEG1, PEG10, H19* and *NNAT*) in fresh and aged oocytes *in vitro* aging with or without cumulus cells for 24 h or 48 h. (**B**) The development of blastocysts from fresh and aged oocytes *in vitro* aging for 24 h after parthenogenetic activation. (**C**) Relative expression of related imprinted genes (*GRB10, IGF2, PEG1, PEG10, H19* and *NNAT*) in blastocysts from fresh and aged oocytes *in vitro* aging for 24 h after parthenogenetic activation. All graphs show mean ± s.e.m. Abbreviations used in this and all subsequent figures: COC, cumulus oocyte complex; DO, denuded oocyte; IVA, *in vitro* aging. a–c: Values without a common letter in their superscripts differ significantly (P < 0.05).

To study the roles of imprinted genes during oocyte aging, several imprinted genes (*GRB10, IGF2, PEG1, PEG10, H19* and *NNAT*) were selected to detect their expression in aged oocytes. Quantification indicated that oocyte aging decreased the expression of *PEG1* and *NNAT* in oocytes aged for 24 h and 48 h and there were few differences between 24 h and 48 h. To make sure whether cumulus cell affected oocyte aging and the expression of imprinted genes, fresh oocytes were aged for 24 h and 48 h *in vitro* with or without cumulus cells. It showed that there were no differences between aged oocytes with or without cumulus cells (Fig. 1A). So we focused the oocytes aged for 24 h *in vitro* without cumulus cells in the following experiments.

It has been proved that oocyte quality determined the embryo’s developmental potential and oocyte aging was a key factor to affect oocyte quality. We found that 45.0% fresh oocytes could develop to blastocyst after parthenogenetic activation, however, there were only 14.0% oocytes aged for 24 h *in vitro* that developed to blastocysts (Fig. 1B). Next, we detected the expression of these imprinted genes in blastocysts from fresh oocytes and oocytes aged for 24 h. The results showed that the expression of *NNAT* was decreased significantly in blastocysts from aged oocytes. Only *IGF2* and *H19* mRNA levels were increased significantly (Fig. 1C).

### DNA methylation pattern of *NNAT* and *H19* in aged oocytes and blastocysts by parthenogenetic activation

**Figure 2:**
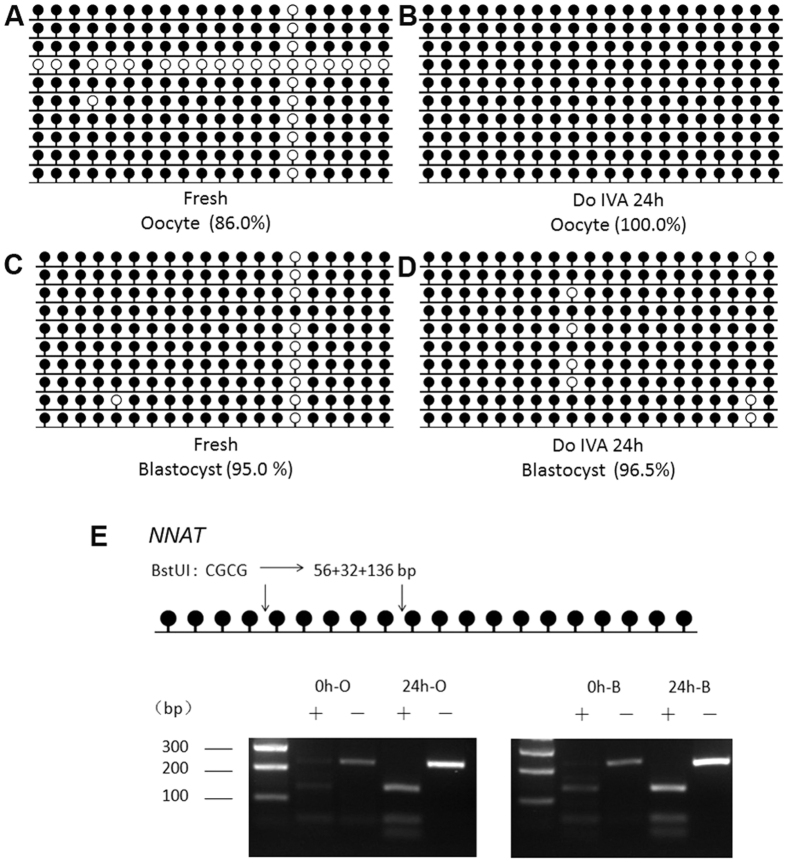
DNA methylation pattern of *NNAT* in aged oocytes and blastocysts by parthenogenetic activation. (**A**) Cytosine methylation profiles of *NNAT* in fresh oocytes. (**B**) Cytosine methylation profiles of *NNAT* in age oocytes *in vitro* aging for 24 h without cumulus cells. (**C**) Cytosine methylation profiles of *NNAT* in blastocysts from fresh oocytes after parthenogenetic activation. (**D**) Cytosine methylation profiles of *NNAT* in blastocysts from aged oocytes *in vitro* aging for 24 h after parthenogenetic activation. (**E**) Overall methylation profiles of the DMRs in fresh and aged oocytes and blastocysts by parthenogenetic activation analyzed by COBRA. Abbreviations used in this and all subsequent figures: 0 h-O, oocyte aged for 0 h (fresh oocyte); 24 h-O, oocyte aged for 24 h, 0 h-B, blastocyst from oocyte aged for 0 h after parthenogenetic activation, 24 h-B: blastocyst from oocyte aged for 24 h after parthenogenetic activation.

Our previous studies have shown that there was close relationship between expression and DNA methylation pattern in imprinted genes during oocyte aging^6^. So we detected DNA methylation pattern of *NNAT* and *H19* in both oocytes aged for 24 h *in vitro* without cumulus cells and their blastocysts by parthenogenetic activation. To detect DNA methylation pattern of *NNAT* and *H19*, we employed both bisulfite DNA sequencing (BSP) and combined bisulfite restriction analysis (COBRA) methods^22^. The data showed that DNA methylation of *NNAT* was increased in aged oocytes comparted to fresh ones. Methylation of *NNAT* in aged and fresh oocytes was 100.0% and 86.0% by BSP, respectively (Fig. 2A,B). To ensure that the sequencing results could reflect the real methylation pattern, we carried out COBRA. We used BstUI to digest the same bisulfite-treated PCR amplification products. The data showed the same results that *NNAT* was hypermethylated in aged oocytes (Fig. 2E). However, there was no difference between blastocysts from fresh oocytes and oocytes aged for 24 h. Methylation of *NNAT* in blastocysts from fresh and aged oocytes were 95.0% and 96.5% by BSP, respectively (Fig. 2C,D).

**Figure 3:**
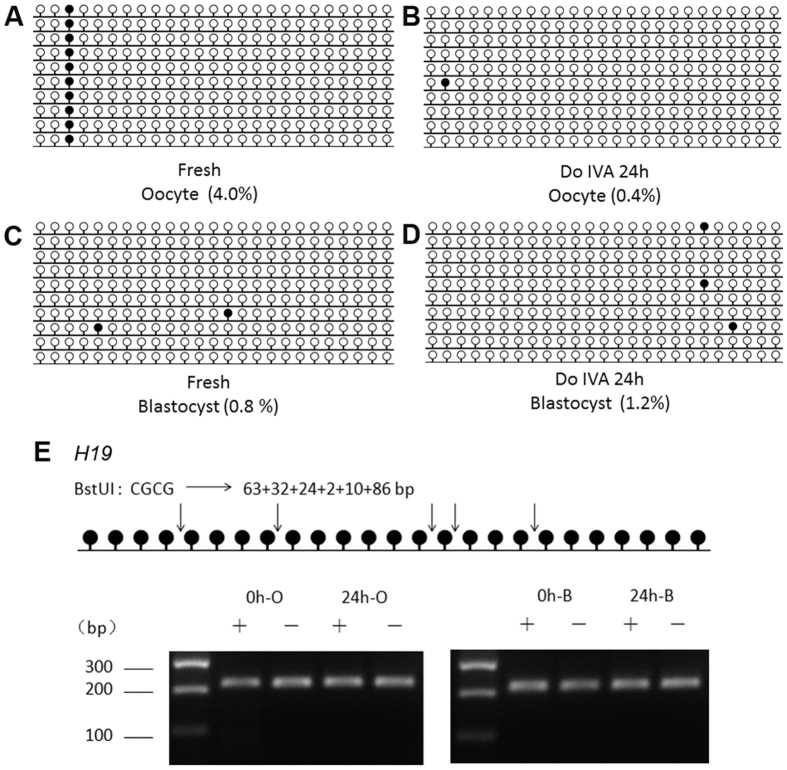
DNA methylation pattern of *H19* in aged oocytes and blastocysts by parthenogenetic activation. (**A**) Cytosine methylation profiles of *H19* in fresh oocytes. (**B**) Cytosine methylation profiles of *H19* in age oocytes *in vitro* aging for 24 h without cumulus cells. (**C**) Cytosine methylation profiles of *H19* in blastocysts from fresh oocytes after parthenogenetic activation. (**D**) Cytosine methylation profiles of *H19* in blastocysts from aged oocytes *in vitro* aging for 24 h after parthenogenetic activation. (**E**) Overall methylation profiles of the DMRs in fresh and aged oocytes and blastocysts by parthenogenetic activation analyzed by COBRA.

*NNAT* was a maternal imprinted gene, so we detected another paternal imprinted gene *H19*. The expression of *H19* in aged oocytes for 24 h *in vitro* had no significant change; however, its expression was increased in blastocysts from oocytes aged for 24 h significantly (Fig. 1C). BSP and COBRA data showed that oocyte aging had no effects on DNA methylation pattern of *H19* in both oocytes and parthenogenetic blastocysts (Fig. 3).

### Glucose transport was impaired in aged oocytes

**Figure 4:**
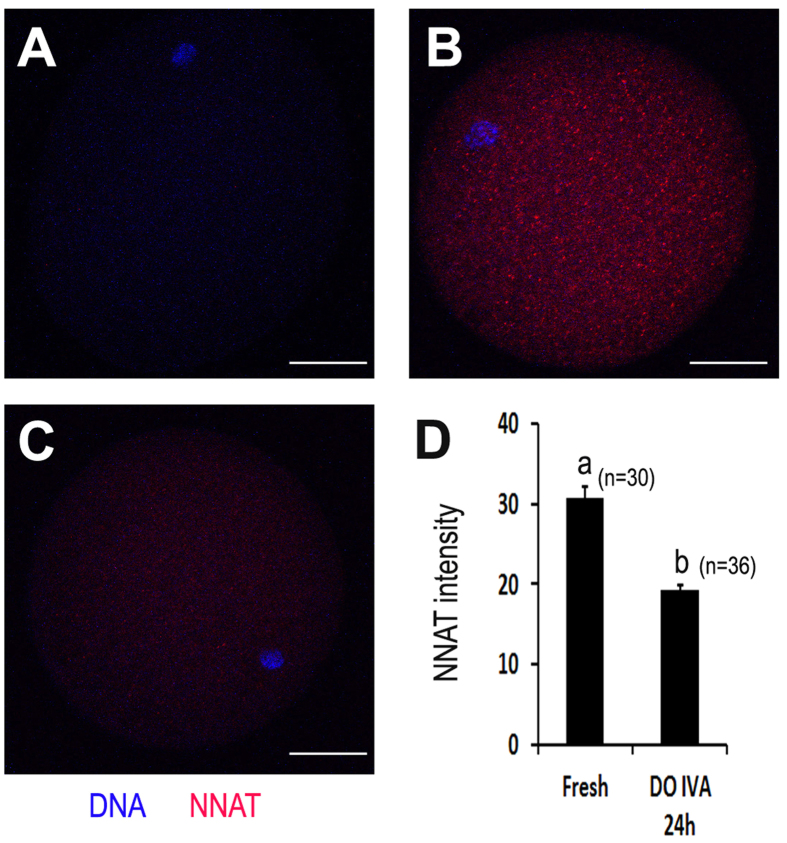
Oocyte aging decreased the expression of NNAT at protein level in porcine oocytes. Confocal micrographs of porcine oocytes show NNAT expression on protein level (red NNAT, blue DNA). (**A**) Negative control (staining without primary antibody), (**B**) NNAT in fresh oocytes, (**C**) NNAT in aged oocytes *in vitro* aging for 24 h without cumulus cells. (**D**) Quantification of NNAT expression in fresh oocytes and aged oocytes *in vitro* aging for 24 h without cumulus cells. The graph shows mean ± s.e.m. a–b: Values without a common letter in their superscripts differ significantly (P < 0.05). Scale bars, 20 μm.

**Figure 5:**
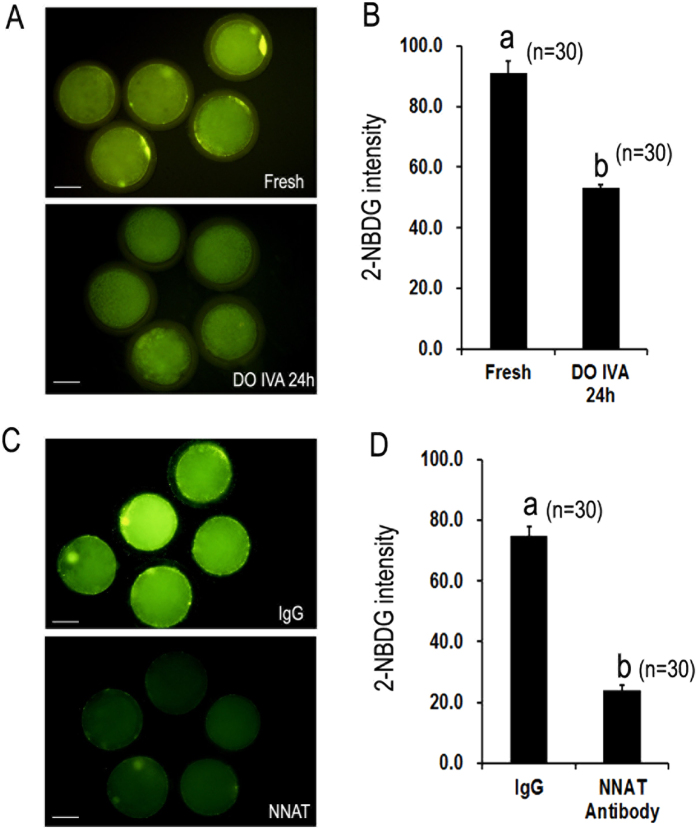
The effects of oocyte aging and NNAT inhibition on glucose transport in porcine oocytes. Glucose transport was tracked by a fluorescent glucose analog, 2-NBDG. (**A**) Glucose transport in fresh oocytes and aged oocytes *in vitro* aging for 24 h without cumulus cells. (**B**) Quantification of glucose transport in fresh and aged oocytes. The graph shows mean ± s.e.m. (**C**) Glucose transport in fresh oocytes injected with rabbit immunoglobulin G (IgG) and NNAT antibody. (**D**) Quantification of glucose transport in fresh oocytes injected with IgG and NNAT antibody. The graph shows mean ± s.e.m. a–b: Values without a common letter in their superscripts differ significantly (P < 0.05). Scale bars, 40 μm.

Based on the above findings, *NNAT* mRNA levels were reduced in both aged oocyts and blastocysts by parthenogenetic activation. So we detected whether the level of NNAT protein was decreased in aged oocytes or not by IF. The results showed that the expression of NNAT in aged oocytes was declined to about one third of that in fresh oocytes (Fig. 4). Energy metabolism is very important for optimal development of oocytes and glucose transport provids enough glucose for energy metabolism in oocytes. To determine whether oocyte aging affected glucose transport, 2-NBDG was used to track glucose transport in oocytes. The results showed that 2-NBDG uptake was reduced with the time of aging. In fresh oocytes, they had a considerable potential to uptake glucose, however, the fluorescence intensity of 2-NBDG in aged oocytes was significantly decreased compared to fresh oocytes (91.0 ± 4.1 vs. 53.3 ± 1.0, p < 0.05), suggesting that oocyte aging blocked glucose transport in oocytes (Fig. 5A,B).

### NNAT inhibition decreased glucose transport in aged oocytes

NNAT has been found to upregulate glucose transport 1(GLUT-1) by activating PI3K-Akt2 signaling pathway^11^. To determine whether NNAT affected glucose transport in oocytes, we injected NNAT antibody into the cytoplasm of fresh MII oocytes to block the function of NNAT. The results showed that the transportation of glucose was decreased significantly in oocytes injected with NNAT antibody. The fluorescence intensity of 2-NBDG in oocytes injected with NNAT antibody was significantly decreased compared to oocytes injected with IgG (74.8 ± 3.1 vs. 23.9 ± 1.8, p < 0.05), suggesting that oocyte aging blocked glucose transport in oocytes (Fig. 5C,D). These data showed that the loss of NNAT affected glucose transport in aged oocytes.

## Discussion

In this study, we used porcine oocytes as a model to demonstrate an imprinted gene, *NNAT*, regulates oocyte aging by controlling glucose transport in porcine. Alternations in glucose transport induced abnormal glucose metabolism, which broke the metabolic balance in oocytes and affected the development of oocytes after fertilization. NNAT could activate PI3K-Akt2 signaling pathway to upregulate glucose transport 1(GLUT-1)^11^. Our observation is important to indicate the relationship between oocyte aging and glucose transport, which provides a promising approach to delay oocyte aging.

Cumulus cells accelerate oocyte aging in mouse oocytes^23^. In porcine oocytes, whether cumulus cells accelerate oocyte aging has not been investigated. In our studies, we selected several imprinted genes (*GRB10, IGF2, PEG1, PEG10, H19* and *NNAT*), maternal genes (*BRG1, ZAR1, BMP15* and *TET3*, Fig. S1) and pluripotent gens (*POU5F1, SOX2* and *CDX2*, Fig. S2) and compared their expression in fresh oocytes, oocytes aged for 24 h and 48 h *in vitro* with or without cumulus cells. To avoid the effects of variation in the developmental potential of embryos by different source of sperm^24^, we detected their expression in parthenogenetic blastocysts from fresh and aged oocytes. It showed that different aging time and cumulus cells had no effects on gene expression in porcine oocytes. And only *PEG1* and *NNAT* mRNA level was declined in both aged oocytes and blastocyst. PEG1 has been studied in aged mouse oocytes, so our studies were focused on *NNAT* in the following experiments.

Epigenetic modification is proved to maintain normal patterns of DNA methylation or histone modification and stabilize gene expressions which are necessary for embryo development. This process is highly involved in many events during early embryo preimplantation development, including maternal-to–zygotic transition (MZT), compaction and the first two distince cell lineages (inner cell mass (ICM) and trophectoderm (TE)). Abnormal epigenetic modification, like impairing DNA methylation of imprinted genes, induces the damage to embryo and leads to deleterious influence on the development of embryos^25^. In our studies, we found that oocyte aging disturbed the expression of several imprinted genes dramatically and parthenogenetic blastocyst percentage from aged oocytes was significantly decreased (Fig. 1A,C), suggesting that oocyte aging impaired its developmental potential by affecting the patterns of DNA methylation of imprinted genes. The establishment of parent-specific methylation imprints during oogenesis could pass to their offspring. However, their DNA methylation pattern was affected by many factors, like stress, environment, and age. Our previous studies showed that Snrpn methylation was lost in aged mouse oocytes^6^ and this loss affected the expression of some special genes in placentas or fetus^26^. The whole DNA methylation pattern or specific CpG sites methylation pattern of genes altered their expression^27^. Our data suggest that the methylation level of *NNAT* is higher in oocytes aged for 24 h compared to fresh oocytes; however, there is no difference in their parthenogenetic blastocysts. The digestion results are consistent with the sequencing results (Fig. 2E). When we analyze 20 CpG sites in the CpG island of the *NNAT* promoter region, we find a very special CpG site, site 15, whose methylation levels are very low in fresh oocytes and their parthenogenetic blastocysts. In contrast, the methylation levels of the same site are extremely higher in aged oocytes and their parthenogenetic blastocysts. We speculate that specific CpG sites methylation pattern of *NNAT* will be more important for expression. Further studies are needed to prove their relationships. To exclude the special effects of *NNAT*, we also detect another imprinting gene, *H19*. The data shows that there is no difference in mRNA and DNA methylation level in both fresh and aged oocytes (Fig. 3).

Glucose transport 1(GLUT-1) was expressed in oocytes or embryos and controlled their glucose uptake in human, bovine and mouse^28^,^29^,^30^. It was proved that glucose was absorbed by cumulus cells by the GLUT system and then transferred into the oocyte through gap junctions in mouse oocytes^31^. However, we found that glucose could be transported into oocyte directly without cumulus cells in porcine oocytes and this transport was declined in age oocytes. NNAT regulated GLUT-1 by activating PI3K-Akt2 signaling pathway^11^. Our data showed that 68.0% glucose transport was blocked when fresh oocytes were injected with NNAT antibody, which suggested that NNAT inhibition would disturb GLUT-1 to uptake glucose and affect oocyte quality. Oxidative stress (OS) caused oocyte apoptosis and cytoskeleton alterations by BCL2 during oocyte aging. However, low temperature and antioxidants could overcome the deleterious effects induced by OS^32^,^33^. As proved that pyruvate involved mitochondrial electron transport and MCTs and regulated both the intracellular redox status and energy supply to inhibit oocyte aging^9^. Our data showed that the decrease of NNAT activity impaired partial transported glucose, which may affect the production of antioxidants, like pyruvate. However, retained NNAT activity and limited glucose transport were still sufficient for oocyte survival and maintained its weak developmental potential. In summary, we first reported that *NNAT* was hypermethylated in aged porcine oocytes and this hypermethylation may lead to decreasing its expression in both aged oocytes and blastocysts by parthenogenetic activation in this study. We also found that glucose transport was blocked in aged oocytes and fresh oocytes injected with NNAT antibody. These results suggest that the expression of *NNAT* is declined by altering DNA methylation, which affects oocyte quality by decreasing the ability of glucose transport in aged oocytes. The data obtained not only have contributed to our understanding of the mechanism of oocyte aging but also provided important information that could potentially be used to control oocyte aging in related animal or clinical assisted reproductive technology.

## Additional Information

**How to cite this article**: Gao, Y.-Y. *et al*. Oocyte aging-induced *Neuronatin* (*NNAT*) hypermethylation affects oocyte quality by impairing glucose transport in porcine. *Sci. Rep.* **6**, 36008; doi: 10.1038/srep36008 (2016).

**Publisher’s note:** Springer Nature remains neutral with regard to jurisdictional claims in published maps and institutional affiliations.

## Supplementary Material

Supplementary Information
