# Supplementary material for: Oocyte aging-induced Neuronatin (NNAT) hypermethylation affects oocyte quality by impairing glucose transport in porcine
Source: Sci Rep. 2016 Oct 26;6:36008. doi: 10.1038/srep36008 (PMC5080544; doi:10.1038/srep36008)
Supplement: Supplementary Information [file srep36008-s1.pdf]

Oocyte aging-induced *Neuronatin (NNAT)* hypermethylation affects oocyte quality by impairing glucose transport in porcine.

Ying-Ying Gao[a], Li Chen[a], Tao Wang[a], Zheng-Wen Nie[a], Xia Zhang[b,c] and Yi-Liang Miao[a, c,]*

([a]Key Lab of Agricultural Animal Genetics, Breeding, and Reproduction of Ministry of Education, College of Animal Science and Technology, Huazhong Agricultural University, Wuhan, 430070; [b]College of Veterinary Medicine, Huazhong Agricultural University, Wuhan 430070; [c]The Cooperative Innovation Center for Sustainable Pig Production, Wuhan 430070, China)

Running title: NNAT and oocyte aging

Keywords: oocyte aging, NNAT, porcine, glucose transport

*Corresponding author: Yi-Liang Miao, 1 Shizishan St, Hongshan District, Wuhan, Hubei Province 430070, China ; Phone: +86-151-7238-1596; Email: miaoyl@mail.hzau.edu.cn

Supplemental Figure legends:

Figure S1:

The expression of related maternal genes in fresh and aged oocytes and blastocysts by parthenogenetic activation. A. Relative expression of related maternal genes (*BRG1, ZAR1, BMP15* and *TET3*) in fresh and aged oocytes *in vitro* aging with or without cumulus cells for 24 h or 48 h. **B.** Relative expression of related maternal genes (*BRG1, ZAR1, BMP15* and *TET3*) in blastocysts from fresh and aged oocytes *in vitro* aging for 24 h after parthenogenetic activation. All graphs show mean ± s.e.m. Abbreviations used in this and all subsequent figures: COC, cumulus oocyte complex; DO, denuded oocyte; IVA, *in vitro* aging. a–d: Values without a common letter in their superscripts differ significantly ($P < 0.05$).

Figure S2:

The expression of related pluripotent gens in fresh and aged oocytes and blastocysts by parthenogenetic activation. A. Relative expression of related pluripotent gens (*POU5F1, SOX2* and *CDX2*) in fresh and aged oocytes *in vitro* aging with or without cumulus cells for 24 h or 48 h. **B.** Relative expression of related pluripotent gens (*POU5F1, SOX2* and *CDX2*) in blastocysts from fresh and aged oocytes *in vitro* aging for 24 h after parthenogenetic activation. All graphs show mean ± s.e.m. a–b: Values without a common letter in their superscripts differ significantly ($P < 0.05$).

Table S1 Primer sequences for qRT-PCR

Gene	Primer sequence 5'-3'	Gene Access no./reference	Length (bp)
GRB10	F:CAAAGGCATTTGTCCTCACA	NM_001134965.1	133
	R:AGCTGGATCAAGTCGGAGAA		
IGF2	F:CTCGTGCTGCTCGTCTTCTT	X56094.1	105
	R:GACAAACTGGAGGGTGTCCA		
PEG1	F:CCAGTGAATCCCTACCCAGA	NM_001128471.1	152
	R: AAGGAGTTGATGAAGCCCATA		
PEG10	F:CGTCTTTGAAGACCCTCAGC	DQ323403.3	128
	R:CGTTCCAATCCAGATCCTGT		
H19	F:CTCAAACGACAAGAGATGGT	Park et al.[1]	122
	R:AGTGTAGTGGCTCCAGAATG		
NNAT	F:CGACAATACCAGATTCCTTC	Park et al.[1]	138
	R:CTTGGTCCAGATCAGAATGT		
POU5F1	F:TTTGGGAAGGTGTTCAGCCAAACG	Zhao et al. [2]	198
	R:TCGGTTCTCGATACTTGTCCGCTT		
SOX2	F:TGTCGGAGACGGAGAAGCG	Zhao et al. [2]	94
	R:CGGGGCCGGTATTTATAATCC		
CDX2	F:CTTGGAGCTGGAGAAGGAGT	GU017420.2	136
	R:TTTCCTCTCCTTCGCTCTGC		
BRG1	F:GGAAAGAGAAGGCCCAGGAT	XM_013994692.1	138
	R:CTTCCTCACTGCCACTTCCT		
ZAR1	F:AACCCTTATCGTGTGGAGGA	NM_001129956.1	128
	R:CCTTTGCATCTCCCACACAA		
BMP15	F:TGGTTAATGGAGCAAGGCCT	NM_001005155.1	130
	R:GCTAGGTGAAGTTGATGGCG		
TET3	F:AGCTGAGCACTCCTGAGAAG	XM_003125027.5	142
	R:CTAAAGTGGTTCTGCGGCTC		

Table S2 Primer sequences for nest-PCR

Gene	Primer sequence 5'-3'	reference	Length (bp)
H19-BSP-out	F:TATGTTTAGGGGTGATAAAAGT	Park et al. [3]	405
	R:CCCCACTTCTACAATTCAAC		
H19-BSP-in	F:AGGTGTTATTTTGTTTGTTGGT		217
	R:ATAAAATAACCTAAAAAAACTCAA		
NNAT-BSP-out	F:ATAGTAGGTGTTTAGTGGAGAG	Chen et al. [4]	748
	R:ATAATCACCGAATATCTACCCTAT		
NNAT-BSP-in	F:TGTGTTAGGTAGTTTGTTGGAGAGA		224
	R:CTCCCAAACCCTAATAAATCTTCTT		

[1] Park CH, Uh KJ, Mulligan BP, Jeung EB, Hyun SH, Shin T, et al. Analysis of imprinted gene expression in normal fertilized and uniparental preimplantation porcine embryos. PloS one 2011;6:e22216.
[2] Zhao M, Isom SC, Lin H, Hao Y, Zhang Y, Zhao J, et al. Tracing the stemness of porcine skin-derived progenitors (pSKP) back to specific marker gene expression. Cloning and stem cells 2009;11:111-22.
[3] Park CH, Kim HS, Lee SG, Lee CK. Methylation status of differentially methylated regions at Igf2/H19 locus in porcine gametes and preimplantation embryos. Genomics 2009;93:179-86.
[4] Chen X, Wang T, Lv Q, Wang A, Ouyang H, Li Z. DNA methylation-mediated silencing of neuronatin (NNAT) in pig parthenogenetic fetuses. Gene 2014;552:204-8.
